# New open conformation of SMYD3 implicates conformational selection and allostery

**DOI:** 10.3934/biophy.2017.1.1

**Published:** 2016-12-20

**Authors:** Nicholas Spellmon, Xiaonan Sun, Wen Xue, Joshua Holcomb, Srinivas Chakravarthy, Weifeng Shang, Brian Edwards, Nualpun Sirinupong, Chunying Li, Zhe Yang

**Affiliations:** 1 Department of Biochemistry and Molecular Biology, Wayne State University School of Medicine, Detroit, Michigan, USA; 2 Center for Synchrotron Radiation Research and Instrumentation and Department of Biological and Chemical Sciences, Illinois Institute of Technology, Chicago, Illinois, USA; 3 Nutraceuticals and Functional Food Research and Development Center, Prince of Songkla University, Hat-Yai, Songkhla, Thailand; 4 Center for Molecular and Translational Medicine, Georgia State University, Atlanta, GA, USA

**Keywords:** lysine methyltransferase, MYND- and SET-domain containing protein, epigenetics

## Abstract

SMYD3 plays a key role in cancer cell viability, adhesion, migration and invasion. SMYD3 promotes formation of inducible regulatory T cells and is involved in reducing autoimmunity. However, the nearly “closed” substrate-binding site and poor in vitro H3K4 methyltransferase activity have obscured further understanding of this oncogenically related protein. Here we reveal that SMYD3 can adopt an “open” conformation using molecular dynamics simulation and small-angle X-ray scattering. This ligand-binding-capable open state is related to the crystal structure-like closed state by a striking clamshell-like inter-lobe dynamics. The two states are characterized by many distinct structural and dynamical differences and the conformational transition pathway is mediated by a reversible twisting motion of the C-terminal domain (CTD). The spontaneous transition from the closed to open states suggests two possible, mutually non-exclusive models for SMYD3 functional regulation and the conformational selection mechanism and allostery may regulate the catalytic or ligand binding competence of SMYD3. This study provides an immediate clue to the puzzling role of SMYD3 in epigenetic gene regulation.

## 1. Introduction

SMYD3 belongs to a special class of protein lysine methyltransferases containing SET and MYND domains [1]. The SET is a catalytic motif responsible for lysine methylation. The MYND is a protein-protein interaction module involved in transcriptional cofactor recruitment. SMYD3 is overexpressed in more than 15 types of cancers such as breast cancer, colon cancer, prostate cancer, lung cancer and pancreatic cancer [1–4]. Overexpression of SMYD3 often correlates with poor prognosis and its knockdown inhibits tumor growth [2,4]. Therefore, drug intervention of SMYD3 may be beneficial to the fields of cancer. SMYD3 is involved in tumorigenesis through methylation of histone and non-histone proteins. Histone methylation regulates gene expression and methylation of non-histone proteins can impact biochemical and cellular functions of the targets [1,3,4]. SMYD3 may directly or indirectly methylate histone H3K4, H4K20 and H4K5 [2,5,6]. Through these methylations, SMYD3 is involved in tumor cell viability, adhesion, migration and invasion. SMYD3 upregulates multiple cancer genes through H3K4 trimethylation. These include the telomerase reverse transcriptase (TERT), oncogenic c-Met, matrix metalloproteinase 9 (MMP-9), androgen receptor, myosin regulatory light chain 9 (MYL9) and retinoblastoma protein-interacting zinc finger gene 1 (RIZ1) [1,7–11]. SMYD3 targets Cyclin D2 through H4K20 trimethylation and contributes to a more aggressive phenotype of prostate cancer [5]. H4K5 methylation by SMYD3 provides a potential new link between chromatin dynamics and neoplastic disease [6]. SMYD3 methylates three non-histone proteins: MAP3K2, vascular endothelial growth factor receptor-1 (VEGFR1) and AKT1. Methylation of MAP3K2 prevents PP2A phosphatase, a key negative regulator of the MAP kinase pathway, from binding to MAP3K2 [3]. Methylated MAP3K2 links SMYD3 to Ras-driven cancer promoting cell proliferation and tumorigenesis [3]. VEGFR1 methylation by SMYD3 augments VEGRF1 kinase activity, which is thought to enhance carcinogenesis [12]. Methylation of AKT1 at lysine 14 is essential for AKT1 activation [13]. In addition, SMYD3 was found to promote formation of inducible regulatory T cells and may be involved in reducing autoimmunity [14,15].

SMYD3 in vitro methyltransferase activity is not fully consistent with its cellular activity. SMYD3 only weakly methylates H3K4 in vitro but its cellular methyltransferase activity has been associated with H3K4 trimethylation at many genes [2,3]. This functional inconsistency has hindered further understanding of the role of SMYD3 in epigenetic gene regulation [3,6]. However, poor in vitro activity can be partly explained by the crystal structures [16]. SMYD3 has a closed conformation and a direct lobe-lobe interaction forms a cap over the substrate-binding site. Though this cap structure does not prevent substrate binding, the resulting narrow opening to the active site cavity could potentially affect the substrate binding competence of SMYD3 and thereby the catalytic activity [17]. SMYD3 in vitro activity can be enhanced by Hsp90 and DNA binding [2,18]. The Hsp90 binding site has been mapped to a TPR-like C-terminal domain (CTD) [19]. Due to the closed conformation, the predicted Hsp90 binding site is half-buried and therefore the question remains how Hsp90 binds to SMYD3 and enhances its activity. The DNA binding site was predicted to be located within the zinc-finger MYND domain [18]. However, this domain is indispensable for SMYD enzymatic activities and how SMYD3 activity is regulated by DNA binding remains a puzzle [1]. Here we present an open SMYD3 conformation and both theoretical and experimental evidence that the conformational selection mechanism and allostery may be involved in SMYD3 functional control.

## 2. Materials and Method

### 2.1. Molecular dynamics simulation

Molecular dynamics simulation was performed using NAMD [20]. Initial structure for simulation was the crystal structure of human SMYD3–sinefungin complex (PDB code: 3PDN). Prior to the simulation, this structure was modified by substituting the cofactor analog sinefungin with cofactor S-adenosyl methionine (SAM or AdoMet). The substitution was based on the structural comparison with the SMYD3–SAM complex (PDB code: 5CCL) and the two SMYD3 structures are very similar with a root-mean-squared-deviation (RMSD) of 0.6 Å. The resulting system including the cofactor SAM was parameterized using CHARMM force field (version 36). The net charge of the Zn ions in the structure was set to +2 and the chelating cysteine and histidine residues were deprotonated. The system was solvated inside an orthorhombic box of water molecules with a 13 Å padding in each direction. The system was then neutralized with NaCl at a concentration of 0.15 M. The final system contained 69,749 atoms. Simulation was performed with a 1 fs time step. Particle Mesh Ewald was used to treat long-range electrostatic interactions and a cutoff of 12 Å was used for non-bonded interactions. Periodic Boundary Conditions were applied during the simulation. The simulation was started with 2,000 steps of energy minimization. The first half of the minimization had harmonic restraints on the protein and the second half unrestrained minimization. The minimized structure was then slow heated from 0 to 300 K over 300 ps. At each integration step velocities were reassigned and the temperature was incremented by 0.001 K. The heated structure was then equilibrated for 300 ps and velocities were rescaled to 300 K at every integration step. The system was further equilibrated using Langevin dynamics for 300 ps at constant temperature (300 K) and pressure (1 bar). The production run was performed in the NVE (microcanonical) ensemble at 300 K. The total simulation time was 50 ns and coordinates were recorded every 1 ps.

### 2.2. Principal component analysis

Principal component (PC) analysis was performed using Bio3D [21]. The entire 50 ns trajectory of 50,000 frames was used in the analysis. The overall translational and rotational motions in the trajectory were eliminated by least squares fitting to the first frame. A 3 N × 3 N covariance matrix was generated using Cartesian coordinates of Cα atoms. Diagonalization of the covariance matrix generated 3 N eigenvectors, each having a corresponding eigenvalue. The trajectory was projected onto a particular eigenvector to reveal concerted motions. Clustering of the trajectory in the PC space was performed using *k*-means algorithm. *k*-means partitions the observations into *k* clusters by minimizing the mean squared distance from each observation to its nearest cluster center. The number of clusters was chosen based on the “elbow criteria”. At a cluster count of two the BSS/TSS (Between-group Sum of Squares/Total Sum of Squares) ratio is 79.8%. The PC analysis-based free energy landscapes were produced by Carma [22]. The domain motions along the PC axes were analyzed using the VMD plugin Hingefind [23].

### 2.3. Temporal analysis of structural attributes

Temporal changes of structural attributes including hydrogen bonds, salt-bridges, solvent accessible surface area (SASA), Phi and Psi were analyzed using the VMD plugin Timeline [24]. Hydrogen bonds were calculated with a distance cutoff of 3.2 Å and angle cutoff of 20°. Salt-bridges were calculated with a distance cutoff of 3.5 Å. SASA was calculated using a radius extension of 1.4 Å. The calculations were performed every 25 ps.

### 2.4. Running cross correlation

Residue-pair-wise cross-correlation coefficients were calculated with Bio3D. Running cross correlation (RCC) was calculated using an in-house code. The first element of RCC was obtained by taking the CC of the initial fixed subset of the trajectory. Then the subset was modified by shifting forward: excluding the first frame of the original subset and including the next frame following this subset in the trajectory. This created a new subset of frames, which was used to calculate the next CC. This process was repeated over the entire trajectory. RCC was a plot of the CC against the middle point of the CC time window. Inter-residue RCC deviation map was a heat-map of the standard deviation (σ) of residue-pair-wise RCC. σ was calculated for each RCC; the heat-map represents the magnitude of σ.

### 2.5. Dynamical network analysis

Dynamical network analysis was done in VMD according to previous protocols [24,25]. Each amino acid in the network was represented by one node and SAM by three nodes. Amino acid nodes were centered on Cα atoms and SAM nodes were located at atoms Cα, C4’ and N9. The edges between nodes were drawn if the residues were within a cutoff distance of 4.5 Å for at least 75% of the trajectory. The edge distances were derived from pairwise correlations which define the probability of information transfer across the edge. Correlations were calculated from the trajectory by the program Carma [22]. The community substructure of the network was obtained using the Girvan-Newman algorithm. Nodes in a community have more and stronger connections within that community than the nodes in other communities.

### 2.6. Targeted molecular dynamics

Targeted molecular dynamics (TMD) simulation was performed with NAMD. The initial and target structures used for simulation were the most dissimilar structures along the PC1 axis in the full-trajectory PCA (see above). During simulation, all heavy atoms in the CTD were guided towards the final target structure by steering forces. The force on each atom was given by the gradient of the potential: *U_TMD_* = ½ * *k* * (*RMS*(t)−*RMS*_0_(t))^2^, where *RMS*(t) was the instantaneous best-fit RMS distance of the current coordinates from the target coordinates, *RMS*_0_(t) was the preset RMSD value for the current time step and the force constant *k* was 200 kcal·mol^−1^·Å^−2^. Other simulation parameters were the same as those used in the above conventional molecular dynamics simulation.

### 2.7. Protein expression and purification

Human SMYD3 was essentially expressed and purified as previously described [16,26]. In brief, SMYD3 was cloned with a His_6_-SUMO tag in a pCDF-SUMO vector. Clones were inoculated in LB media and grew until an OD_600_ reached 0.4–0.6. Cells were induced with 0.1 mM isopropylthio-β-D-galactoside (IPTG) and grown overnight at 15 °C. Cells were harvested and lysed using a French Press. Lysate was spun down and the supernatant was collected for purification. The His_6_-SUMO-SMYD3 was captured with a Ni^2+^-affinity column and the His_6_-SUMO tag was removed by yeast SUMO protease 1. Native protein was separated after running through a second Ni^2+^ column. Finally, SMYD3 was further purified by a size exclusion column in 20 mM Tris pH 8.0, 150 mM NaCl, 3% glycerol and 2 mM Tris(2-carboxyethyl) phosphine (TCEP).

### 2.8. Small angle X-ray scattering

Small angle X-ray scattering (SAXS) data were collected at BioCAT beamline at Argonne National Laboratory. Solution conditions were 20 mM Tris pH 8.0, 150 mM NaCl, 3% glycerol and 2 mM TCEP. All measurements were made at 25 °C using a 100 µL capillary flow-cell. Scattering data were collected at two SMYD3 concentrations: 1.2 and 7.7 mg/mL. Five frames with a 1s exposure were taken and data were averaged and subtracted from averaged buffer frames. Low and high concentration data were merged based on an aligned middle *q* region to generate a single scattering curve with a *q* range of 0.0042–0.39 Å^−1^. Radius of gyration (Rg) values were calculated using the Guinier approximation [27]. The distribution function of interatomic distances within SMYD3, *P(r)*, was estimated from the scattering data using the GNOM algorithm [27]. *Ab initio* dummy atom models were generated using DAMMIN [28]. Normal mode analysis was carried out by SREFLEX [29]. Theoretical scattering curves of SMYD3 structures were calculated with CRYSOL [27].

### 2.9. Statistical analysis

Significance of mean differences for continuous data was evaluated by two-tailed t-test and circular data (Phi and Psi) by Watson-Williams high concentration F test. Association between continuous data was measured with Pearson correlation coefficient (*r*). Association of hydrogen bonds and salt-bridges with conformational states were evaluated by PHI coefficient and association of backbone angels or solvent accessible surface area by point-biserial correlation coefficient. For backbone angles, sine values of angles were used in correlation analysis.

## 3. Results

### 3.1. Conformational transition from the closed to open states

**Figure 1:**
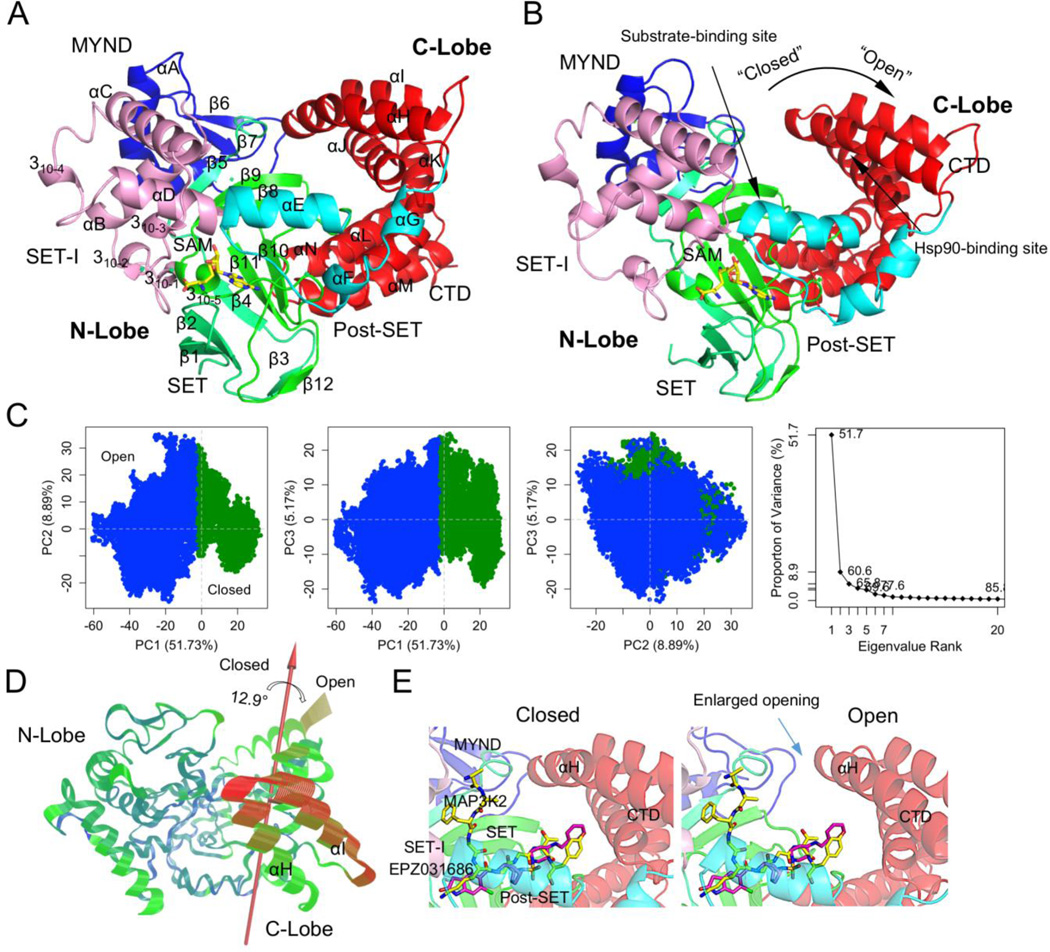
New open conformation of SMYD3. (A) A closed-state and (B) open-state structure. SMYD3 is colored according to domain. Secondary structures are labeled and numbered according to their position in the sequence. (C) Principle component analysis (PCA) of full 50-ns trajectory. Left three, projection of the trajectory onto the planes formed by the first three principle components. Conformers are colored according to the *k*-means clustering. Rightmost, scree plot showing the proportion of variance against its eigenvalue rank. (D) Visualization of the motions along PC1. Color scale from blue, green, to red depicts low to high atomic displacements. (E) Superimposition of the open and closed states with an SMYD3 bound peptide (MAP3K2, yellow) and inhibitor (EPZ031686, purple).

Molecular dynamics (MD) simulation reveals a striking conformational transition of SMYD3 from the closed to open states. The closed state is a crystal structure-like state characterized by a direct lobe-lobe interaction at top of the substrate-binding site (Figure 1A). The open state represents a previously-unidentified new conformational state which lacks the equivalent interaction between the two lobes (Figure 1B). In the closed state, the lobe-lobe interaction involves residues W300 from the C-lobe and S44, V47, V48, Q191 and V193 from the N-lobe (Figure S1A). The interaction includes a hydrogen bond from W300 to S44 and hydrophobic interaction of the W300 side chain with a pocket formed by the aforementioned N-lobe residues. The open state is characterized by the break of the direct lobe–lobe interaction. The W300–S44 hydrogen bond breaks and the side chain of W300 flips out from the small hydrophobic pocket. The substrate-binding site is widened and there is a clear gap between the N- and C-lobes. As a result, the open state shows larger structural difference from the crystal structure (Figure S1B).

The conformational transition can be illustrated by the change in W300–S44 distance. In the closed state W300–S44 maintains a hydrogen bonding distance for most of the time (Figure S2A). In the open state the hydrogen bond breaks and their distance fluctuates between 4.9 Å and 21.2 Å. The distance shows a steep rise during the transition phase and the transition happens in less than 0.3 ns. Therefore, the change in W300–S44 distance can clearly separate the two conformational states. Covariance-based principal component analysis (PCA) further demonstrates the presence of structure-distinct conformational states. The first PC axis alone is sufficient to define two major clusters, one corresponding to the closed state and the other the open state (Figure 1C). The two clusters are well separated along the PC1 axis and the boundary between them is marked by low population of conformers (Figure S2B). This statistically indicates a free-energy barrier for conformational transition. PC1 accounts for more than 50% of overall variance and the motion described by PC1 is a clamshell-like motion between the N- and C-lobes (Figure 1D and S2C). The rotation axis of this motion passes between the two lobes lying at the bottom of the gap between the two lobes. Therefore, this motion essentially depicts an open–closed dynamics and the conformational transition between the closed and open states.

### 3.2. New open ligand-binding-capable conformational state

The new open state may represent a conformational state that facilitates substrate or effector binding to SMYD3. The open state shows an enlarged opening to the substrate binding site which may make it more accessible to a substrate than the closed state (Figure 1E). There is over 35% increase in the accessible volume of the substrate binding cavity in the open state. The first α helix of the CTD (αH) is responsible for the widening and increased accessibility. This helix is involved in the direct lobe–lobe interaction and undergoes a large movement during the transition from the closed to open states (Figure 1D). Because of this movement, the substrate-binding site is widened and more solvent-exposed in the open state.

The predicted Hsp90-binding site also becomes more solvent-exposed in the open state. The C-terminal MEEVD motif of Hsp90 was predicted to bind between αJ and αL at the inner surface of the CTD (Figure S2D) [1,30]. This binding site is structurally similar to the putative TPR peptide-binding site [1]. However, in the closed state the Hsp90-binding site is half-buried due to the direct interaction between αH and the N-lobe. The binding site is further buried due to the lobe-bridging β8–β9 hairpin sitting in front of the binding site. In the open state the distance between the β8–β9 hairpin and Hsp90-binding site becomes significantly larger (Figure S2E) and the volume of the binding site cavity is three times more than that in the closed state. Therefore, the more exposed binding site in the open state may facilitate Hsp90 binding to SMYD3 and provide a mechanistic basis for Hsp90-induced activity enhancement.

### 3.3. Distinct structural characteristics of the closed and open states

**Figure 2:**
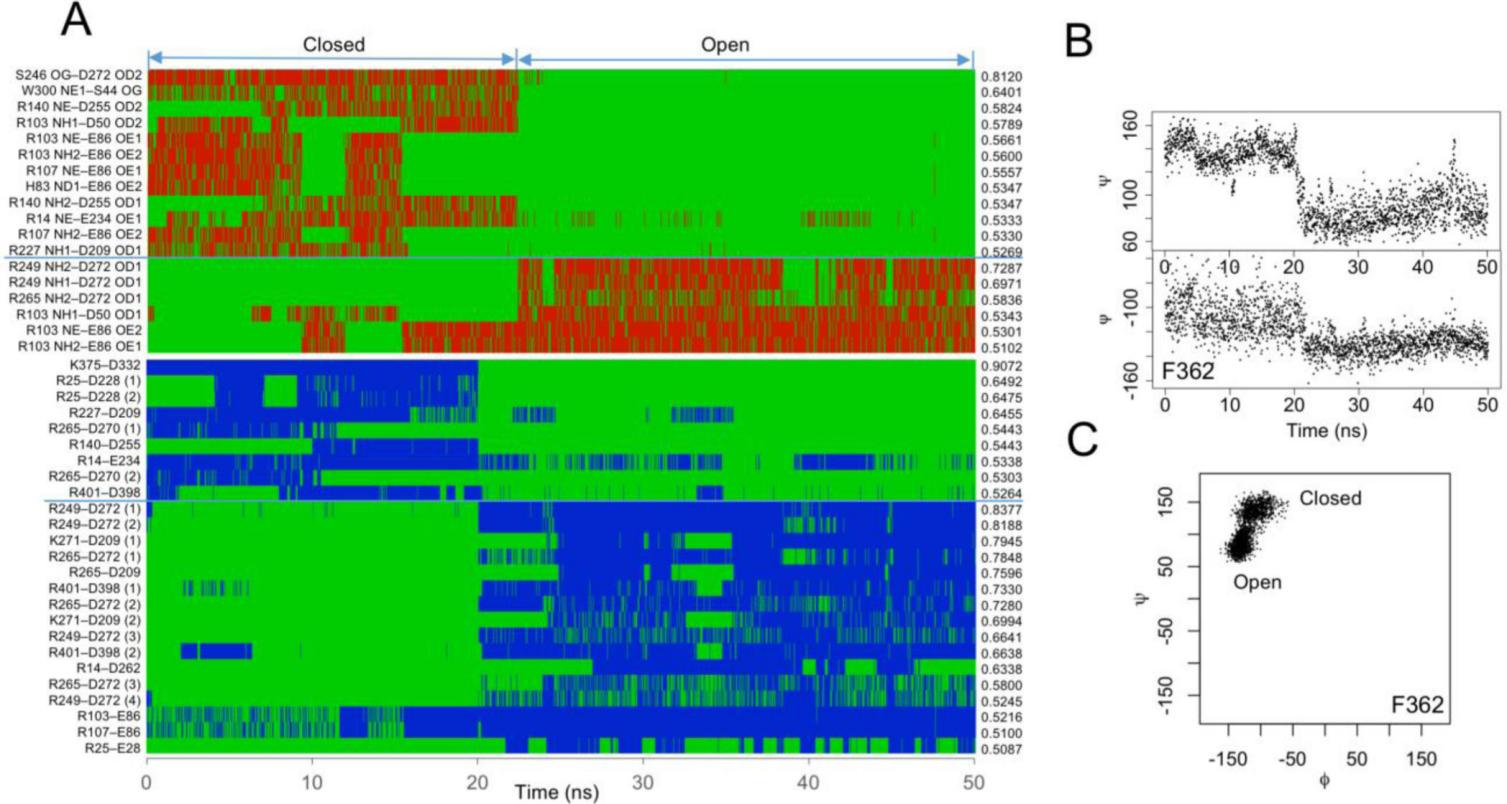
Distinct structural characteristics. (A) Conformational state-correlated hydrogen bonds (top) and salt-bridges (bottom). Red and blue lines indicate the presence of interactions and green lines absence. (B) Torsion angles of F362 as a function of time. (C) Ramachandran plot of F362 trajectory.

The closed and open states show distinct structural characteristics. They are different in hydrogen bonding, salt-bridge, backbone angles and solvent accessible surface area. Hydrogen bonding is different in pattern but not total number (Figure 2A). The closed state has an average of 143.0 hydrogen bonds and open state 142.8. Their difference is not statistically significant (*p* = 0.498). However, there are 18 hydrogen bonds whose time-course pattern shows a significant correlation with the conformational states (*r* > 0.5). Six of them are strongly correlated with the open state and 12 with the closed state including the W300–S44 hydrogen bond. Residue D272 is involved in two conformational state-specific hydrogen bonds. One hydrogen bond (S246–D272) shows the strongest correlation with the closed state and the other (R249–D272) with the open state. D272 is located at the junction between the post-SET and CTD (Figure S3A). In the open state, D272 moves slightly towards the substrate-binding site. The movement breaks its hydrogen bond to S246 and leads to the formation of the hydrogen bond with R249. This indicates that the hydrogen bonds S246–D272 and R249–D272 may be mutually exclusive. The time-course patterns of these two hydrogen bonds show a strong negative correlation (*r* = −0.548).

The numbers of salt-bridges in the closed and open states are significantly different (*p* < 2.2 × 10^−16^). The closed state has 50.4 salt-bridges and open state 54.9. Nine salt-bridges show a significant correlation with the closed state and 16 with the open state (*r* > 0.5) (Figure 2A). The salt-bridge D332–K375 has the strongest correlation with the closed state (*r* = 0.907). This salt-bridge stabilizes the closed state by pulling together the helices αJ and αL of the CTD (Figure S3B). This also contributes to the buried state of the Hsp90-binding site. The salt-bridge D272–R249 shows the strongest correlation with the open state (*r* = 0.838). This correlation is consistent with the open-state-correlated hydrogen bonding between these two residues. The D272–R249 salt-bridge pulls αG towards the substrate-binding site and the pulling squeezes the bottom lobe–lobe interface. The salt-bridge D209–K271 also shows a significant correlation to the open state (*r* = 0.795). However, the direction of the force exerted by this salt-bridge is different from that by the D272–R249 salt-bridge. The D272–R249 exerts the force along the axis of the rotation describing the open-closed lobe–lobe motion. The D209–K271 exerts the force perpendicular to this axis at the opposite surface of the substrate-binding site. The D209–K271 stabilizes the open state by pulling the two lobes outwards.

Many residues show significant differences in the backbone torsion angles. Fifty-five percent of residues are significantly different in Psi and 51% in Phi (*p* < 0.001). Twelve and seven residues show more than 30° differences in Psi and Phi respectively (Table S1). There are 21 residues whose Psi changes show a significant correlation with the conformational states and 12 residues for Phi (*r* > 0.5). Both Psi and Phi of residue F362 show strong correlation with the conformational states (*r_psi_* = 0.983, *r_phi_* = 0.839) (Figure 2B). There are clearly two populations in its Ramachandran plot, one corresponding to the closed state and the other open state (Figure 2C). The neighboring residues of F362 also show large changes in the backbone angles and significant correlation with the conformational states (residues 363–366) (Table S1). These residues are located in a short loop connecting the fourth and fifth helices of the CTD. The changes in their backbone angles are correlated with a twisting motion between those two helices during the conformational transition (see below). Their backbone-angle changes are also correlated with a significant change in F362 interacting network. In the closed state, F362 forms a π-π stacking interaction with Y358 (Figure S3C). In the open state, this interaction is replaced by the stacking interaction with H366. As a result, F362 prevents M242 from interacting with H366. The loss of this interaction may weaken the interaction between the N- and C-lobes near the axis of the rotation describing the open-closed motion.

The SASA of the closed and open states are significantly different (*p* < 2.2 × 10^−16^). Unexpectedly, the closed state is more solvent exposed. The average SASA of the closed state is 116,339.3 Å^2^ and open state 116,250.9 Å^2^. Sixty-eight percent of residues show a significant difference in SASA (*p* < 0.001). Fifty-six percent of these residues are more exposed in the closed state than open state. There are 24 residues whose SASA changes show a significant correlation with the conformational states (*r* > 0.5) (Figure S3D). Seventeen of them are located within the CTD. These include three residues (M335, L344 and Q372) lining the Hsp90-binding site, which are more exposed in the open state; and three residues (C309, A334 and C338) at the interface between the second and third helices of the CTD, which become more buried in the open state. The CTD is a key structural determinant of the closed and open states. The enrichment of residues with the conformational state-specific SASA values reflects the characteristic structural changes in the CTD defining the conformational states.

### 3.4. Different dynamical characteristics

The closed and open states have different dynamical characteristics. They are different in flexibility, cross correlation, interatomic distance fluctuation and dynamical network. The closed state is significantly less dynamical than open state (*p* < 2.2 × 10^−16^). The average atomic displacement of the closed state is 0.81 Å and open state 1.24 Å (Figure S4A). The flexibility of the CTD increases to a larger extent than the N-lobe in the open state. The average ratio of root-mean-square fluctuation (RMSF) of the open to closed states is 1.36 for the N-lobe and 1.83 for CTD. However, the overall fluctuation pattern is not significantly different and the correlation between the two states is 0.753. Most of the residues in both states have a below 1 Å atomic displacement. The least dynamical region is the SET domain in both states. The SET is the catalytic domain responsible for cofactor and substrate binding. Several regions show a notable difference in flexibility. In the closed state the regions around residues W300 and S44 are less dynamical than those in the open state. The two regions interact with each other in the closed state and such interaction appears to restrain their flexibility.

**Figure 3:**
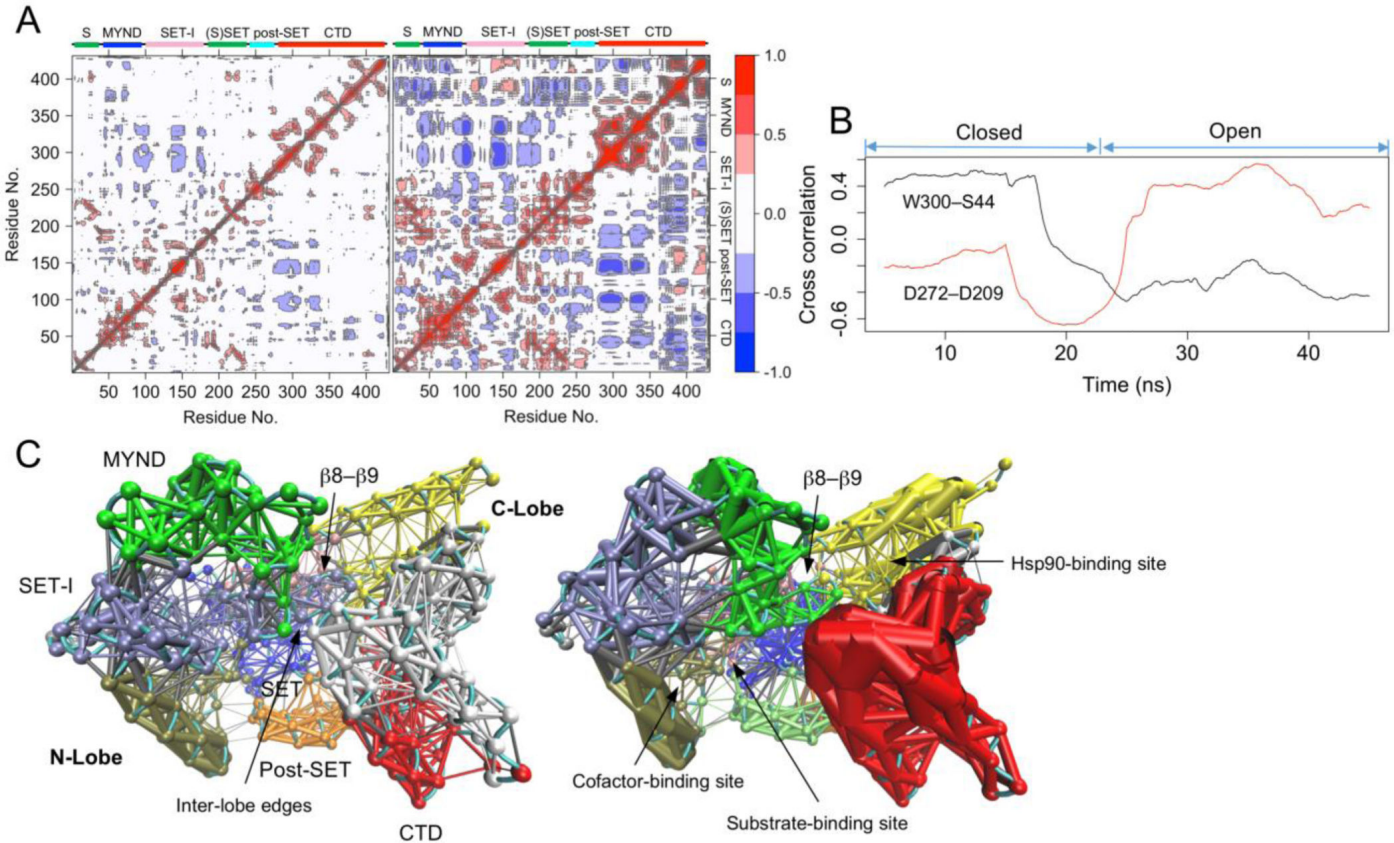
Different dynamical characteristics. (A) Cross-correlation map of the trajectory. Left, the closed state; right, open state. Blue and red indicate negative and positive correlation respectively. (B) Running cross correlation (RCC) of the residue pairs W300–S44 and D272–D209. (C) Dynamical network analysis of the closed (left) and open (right) states. Networks are colored according to communities. Points in the network are nodes and lines between the nodes represent edges. Thicker lines depict the stronger edges or stronger correlations.

Dynamic cross-correlation patterns of the closed and open states are different. The closed state shows a significantly lower level of correlated motions (*p* < 2.2 × 10^−16^). The average correlation coefficients of the closed state and open state are 0.147 and 0.243 respectively. In both states, the SET-I and the first three helices of the CTD show strong negative correlated dynamics and the MYND motion is negatively correlated with the CTD motion (Figure 3A). Such negative correlated dynamics is consistent with the open-closed motion between the N- and C-lobes. The open state shows many additional correlated motions. Among the most notable ones are those between the last three helices of the CTD and many regions across all domains. To quantitatively characterize the dynamical change in correlated motion, we developed the running cross correlation (RCC) method (see Methods). RCC shows a time-course change in cross correlation. It should smooth out short-term fluctuations and highlight longer-term trends or changes. RCC analysis shows that the cross-correlation profile of the residue pair W300–S44 evolves and changes during the simulation (Figure 3B). The motions of W300 and S44 are positively correlated in the closed state when they interact, but change to a negative correlated dynamics after the conformation is transited to the open state. Inter-residue RCC deviation analysis shows that W300–S44 is among the residues pairs with the largest RCC variations (σ = 0.353) (Figure S4B). The largest variation is found between the residue pair D272–D209 (σ = 0.384). These two residues are not in the close proximity but both involved in conformational state-specific hydrogen bonding and salt-bridges (Figure 2A).

The patterns of interatomic distance fluctuation are different between the closed and open states. The closed state shows a significantly lower level of fluctuation (*p* < 2.2 × 10^−16^). The average fluctuation of the closed state and open state are 0.598 Å and 0.880 Å respectively. Both states show large distance variations between the lobes and the variations within the lobes are significantly lower (Figure S4C). The average level of the between-lobe variations of the open state is two times above that of the closed state. This indicates greater distance variability between the N- and C-lobes in the open state. All components of the N-lobe in the open state show significant distance variations with respect to the C-lobe, but only the MYND and SET-I shows large variations in the closed state. The W300–S44 distance deviates about 0.519 Å and 2.075 Å in the closed and open states respectively. This difference is in agreement with the direct interaction of the two residues in the closed state and the break of this interaction in the open state.

Dynamical network and communities are different between the closed and open states. There are ten communities in the closed state and 11 in open state (Figure 3C). The community assignment in both states is roughly correlated with the sequence- and structure-based domain assignment [1]. However, there are significant differences in the ways of partitioning the domains into communities. The most significant difference is found at the CTD. The CTD is split into three major communities in the closed state, whereas in the open state it is split into two. In both states the last three helices of the CTD form a separate community, but its first four helices form a single community in the open state and are split in half along the middle of the helices in the closed state. This indicates that the residues in the first four helices of the CTD have stronger connections in the open state than they do in the closed state. Of note, the predicted Hsp90-binding site is located between the two open-state-CTD communities. Another notable difference in the dynamical networks is found at top of the substrate-binding site. Because of the direct lobe–lobe interaction, there are inter-lobe edges at this location in the closed state; but without the equivalent interaction, the open state has no edge. This indicates that the closed state may possess additional paths for dynamical inter-lobe communication.

### 3.5. Substates

The conformers in the closed and open states can be further clustered into substates. PC analysis shows that both states consist of two major substates but the motions relating the substates are different (Figure S5A). For the closed state, PC1 accounts for nearly one fifth of the overall variance. The major motion along PC1 is a twisting motion of the N-lobe with respect to the C-lobe (Figure S5B). The axis of the twisting passes through the MYND, β8–β9-containing β sheet and middle of the cofactor-binding site. For the open state, PC1 accounts for 38.4% of the overall variance. The major motions along PC1 include a clamshell-like motion between the N-lobe and first four helices of the CTD; and a twisting motion of the last three helices of the CTD with respect to the N-lobe (Figure S5C). The axis of the former rotation aligns with the axis of the motion depicting the conformational transition between the closed and open states (Figure 1B). In the closed state, the PC1-described twisting motion affects the funnel-shape substrate-binding site. The twisting pulls the β8–β9 hairpin and β12–αD loop together and apart. This alters the dimensions of the substrate-binding site. The funnel-shape substrate-binding site has been proposed to contribute to SMYD2 substrate recognition [30]. In the open state, the PC1-described motions affect the dimensions of the inter-lobe gap and the distance between the CTD and β8–β9 hairpin (Figure S5C). As a result, both substrate-binding site and Hsp90-binding site are exposed to different extents in the substates.

### 3.6. Pathway of the conformational transition

Targeted molecular dynamics (TMD) simulation reveals the conformational transition pathway between the closed and open states (Figure S5D). The forward and reverse transitions follow similar structural conversion processes. The two conformational states are interconverted by a reversible CTD rotation. The axis of the rotation passes through the fifth helix (αL) of the CTD parallel to the helical axis. αL is relatively static during the conformational transition. The average RMSF of this helix is 1.7 Å compared to 4.2 Å for the first four helices of the CTD and 2.2 Å for the last two helices. The differences in these RMSFs are significant (*p* < 9.0 × 10^−6^). αL is involved in direct interaction with the β8–β9 hairpin (Figure S5C). This interaction secures αL in position, appears to assist in rotating the CTD around this axis and thereby may contribute to a proper conformational transition between the closed and open states. In agreement with the conventional molecular dynamics (Figure 1E and S2D), TMD also shows that the conformational transition regulates the degrees of exposure of the substrate-binding site and Hsp90-binding site.

### 3.7. Small angle X-ray scattering

**Figure 4:**
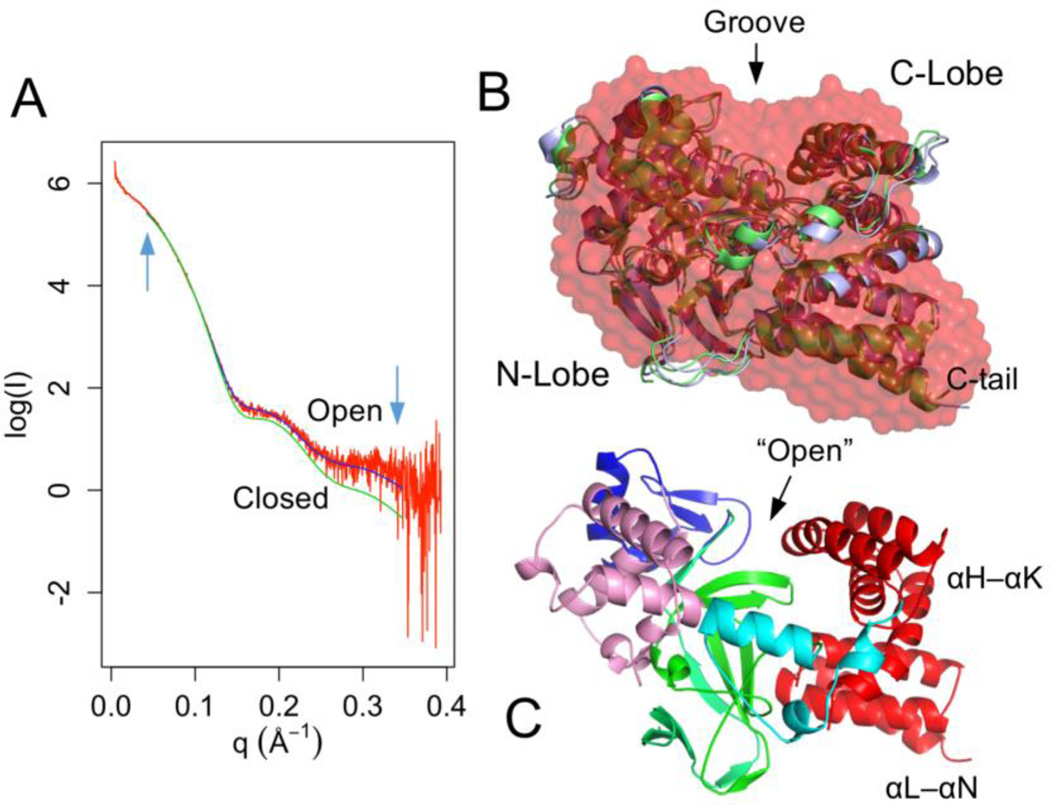
Small-angle X-ray scattering. (A) Experimental scattering curve (red) overlaid with theoretical scattering curves calculated from a closed (green) and open (blue) SMYD3 structure. The *q* range used for model fitting is indicated by arrows. (B) *Ab initio* dummy atom model (red) superimposed with a closed (green) and open (blue) structure. (C) An open structure derived from normal mode analysis.

To provide experimental support for the MD-sampled open state, the solution structure of SMYD3 was characterized using small angel X-ray scattering (SAXS) (Figure 4A). SAXS analysis shows that the radius of gyration (Rg) of SMYD3 is 24.5 Å in solution and D_max_ (maximum particle dimension) 78.0 Å. These values are similar to the Rg (23.2 Å) and D_max_ (77.8 Å) calculated from the crystal structure. The *ab initio* shape modeling shows that the dummy atom model visually matches the crystal structure (Figure 4B). The last three helices of the CTD fits into a slightly protruding envelope and there is a miniature groove between the N- and C-lobe-corresponded regions. However, this dummy model can also be fitted equally well with an open state structure (Figure 4B). This indicates that the low resolution of SAXS model is unable to distinguish between the closed and open states.

The theoretical scattering curve calculated from the crystal structure does not completely fit with the experimental data. The fitted χ^2^ is about 2.68. At the low *q* regions, the fitted curve is in a good agreement with the experimental data, but the high-*q* regions beyond 0.15 Å^−1^ are not being well explained by the fitting (Figure 4A). This suggests that the crystal structure is somewhat different from the solution structure; more strictly, it is different from the average structure of the SMYD3 conformational space. However, the fitting statistics can be improved by normal mode analysis (NMA). NMA probes the large-scale motions in SMYD3 and estimates the structural flexibility to improve agreement with the SAXS data. The best model from the NMA has an improved χ^2^ of 1.72. The CTD in this model undergoes large conformational changes including a twisting motion of the first two helices and an outward-bending motion of its second half (Figure 4C). The lobe–lobe bridging interactions at the W300–S44 interface in this model break. Such a conformation resembles the open state structures sampled in the above MD analysis.

To correlate the MD simulation with the SAXS experiment, the entire MD trajectory was fitted to the experimental data. The average χ^2^ of the trajectory is 3.37 (Figure S6A). The closed state shows significantly lower χ^2^ than the open state (*p* < 2.2 × 10^−16^). The average χ^2^ of the closed state is 2.71 and open state 3.90. This would indicate that the closed state fits better to the SAXS data than the open state. However, the best fitting conformer adopts an open structure. 32% of the open state has a χ^2^ less than the average value of the closed state. This is consistent with normal mode analysis where the open CTD structures show the best agreement with the experimental data (Figure 4C). This also indicates that the combination of all motions in a conformational state determines the results of the experimental data fitting, rather than the open–closed motion alone (Figure 1D and S5). In the open state, the χ^2^ is widely spread with a σ value of 1.63 compared to 0.22 for the closed state (Figure S6A). This is consistent with highly dynamical nature of the open state (Figure S4A). The Rg of the trajectory shows a mixed negative/positive correlation with the χ^2^ (Figure S6B). The open state has larger Rg values than the close state (*p* < 2.2 × 10^−16^). The average Rg for the closed state and open state are 23.2 Å and 23.6 Å respectively. This indicates that the closed-state structures are more compact than the open-state structures. The Rg is strongly negative correlated with the χ^2^ when it is less than 23.4 Å (*r* = 0.612) and changes to a positive correlation at the higher values (*r* = 0.874) (Figure S6B). The negative correlated region samples both closed and open conformers and the population of the closed state in this region is 2.5 times more than that of the open state. However, 75% of the top 1% best fitted conformers adopt an open conformation. This further indicates that some of the open state structures are closer to the average structure of the SMYD3 conformational space.

## 4. Conclusion

SMYD proteins are an exciting field of study as they are linked to many types of cancer-related pathways [2]. Cardiac and skeletal muscle development and function also depend on SMYD proteins opening a possible avenue for cardiac-related treatment [1]. Among SMYD proteins, SMYD3 has received the most attention because of its involvement in epigenetic and non-epigenetic regulation of numerous cancerous genes [1–4]. Due to its tumor-growth-inducing role and association with poor prognosis SMYD3 has emerged as a key target for anti-cancer therapies [31]. However, the biochemical mechanism of SMYD3-mediated methylation remains elusive. The “closed” substrate-binding site and poor in vitro H3K4 methyltransferase activity have led to arguments that SMYD3 is not a histone lysine methyltransferase and the in vivo-associated H3K4 tri-methylation might be catalyzed by other methyltransferases [3,6]. Such arguments have obscured our understanding of the role of SMYD3 in epigenetic gene regulation, where a completely different interpretation of SMYD3 function could result from the arguments: SMYD3 functions as a histone code “writer” defining chromatin states, or only serves to anchor other chromatin-associated proteins through its sequence-specific DNA binding. Here we provide theoretical and experimental evidence that SMYD3 can adopt an open conformation. This new open conformational state is substantially different from the crystal structure-like closed state. The two states are related by a striking clamshell-like motion of the C-lobe with respect to the N-lobe and SMYD3 is transited by this large motion from a ligand binding-incapable state to a binding-capable state. A recent MD study revealed that the CTD can undergo a similar hinge-like motion resulting in expanded substrate binding crevice [32]. In the absence of the cofactor, the CTD samples more open configurations than it does in the presence of the cofactor [32]. It was postulated that the cofactor acts like a key and locks SMYD3 in a closed conformation [32]. However, the present MD study shows that SMYD3 can undergo a spontaneous conformational transition from the closed to open states in the presence of the cofactor. The conformational transition leads to the enlarged opening to the substrate binding site in the open state which could increase histone tail accessibility to the active site cavity and target lysine access channel. This would then provide the mechanism for SMYD3 activity on both H3K4 methylation and H3K4me3 binding. A recent study showed that SMYD3 interacts with H3K4me3 modified histone tails, which facilitates its recruitment to the core promoter regions of most active genes [4].

The conformational transition pathway involves a reversible twisting motion of the CTD and the transition from the closed to open states breaks the top lobe–lobe interface resulting in a more accessible substrate-binding site and Hsp90-binding site. Many structural and dynamical changes are associated with this conformational transition and these changes may either contribute to the transitional process or stabilize the particular conformational states. While the exact portion of each conformational state in solution is unknown, the closed state statistically better fits the experimental data than the open state, but the best fitting conformers adopt an open structure. Nevertheless, the presence of both closed and open states in the conformational ensemble suggests two possible, mutually non-exclusive models for SMYD3 functional regulation. First, a conformational selection mechanism may regulate SMYD3’s ligand binding. In the conformational selection model, the intrinsic dynamics of the protein lead it to spontaneously transition between a stable unbound and a less stable bound conformation. The apo-protein visits the bound state with significant probability and the ligand can bind directly to this conformation shifting the distribution of conformers towards the bound population [33]. Therefore, the open state with the exposed ligand-binding sites suggests that the ligand binding of SMYD3 may be regulated by the conformational selection mechanism. In addition, the highly correlated inter-lobe dynamics in the open state may facilitate SMYD3 promiscuity through the conformational selection mechanism, allowing the structural adaptation to different substrates. The conformational selection mechanism has been shown to be involved in promiscuous ligand binding and this assumes that the protein needs to visit multiple binding conformers capable of binding different ligands [33]. In SMYD3, the inter-lobe dynamics will alter the size of the substrate-binding site. The coupling of the two lobes by the correlated motion might thus offer the specificity and promiscuity for substrate recognition.

Second, our results provide a model for possible allosteric regulation and a population shift between the two conformational states may underlie the functional control of SMYD3. Recent data suggest that allostery can be mediated by transmitted changes in protein dynamics [34]. The binding of an allosteric effector can result in the redistribution of protein conformational ensembles and cause changes in catalytic or ligand binding competence [34]. DNA binding to the N-lobe has been shown to enhance SMYD3 methyltransferase activity [18]. The interaction of SMYD3 with BRD4 mediates the recruitment of transcriptional cofactors at the myostatin gene and regulates skeletal muscle atrophy [8]. SMYD3 interacts with PC4 in tumor cells and such interaction stimulates oncogenic gene expression through deposition of H3K4 tri-methylation [7]. All these interactions are mediated via the MYND domain of SMYD3, but the structural and dynamical consequences of the interaction remain unknown. One possibility is that the interaction may affect the domain dynamics and inter-lobe dynamical correlation. Such an effect could be transduced to other parts of the protein through the edges bridging the dynamical communities and this might in turn cause a population shift between the existing conformational states, thereby modulating active site or binding site geometries. In summary, a detailed study of SMYD3 structure and dynamics is of functional and therapeutic importance. The identification of the open conformational state provides the basis for the conformational selection mechanism and allosteric regulation.

## Supplementary Material

Supplemental
