# Supplementary material for: New open conformation of SMYD3 implicates conformational selection and allostery
Source: AIMS Biophys. Author manuscript; Available in PMC 2017 Jan 1. (PMC5189988; doi:10.3934/biophy.2017.1.1)
Supplement: Supplemental [file NIHMS837256-supplement-Supplemental.docx]

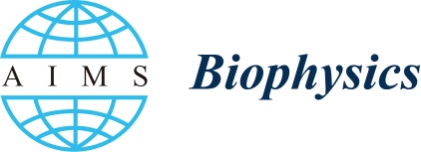
AIMS Biophysics, 4(1): 1-18.

DOI: 10.3934/biophy.2017.1.1
Received: 23 October 2016
Accepted: 12 December 2016
Published: XX December 2016

http://www.aimspress.com/journal/biophysics

***Research article***

**New open conformation of SMYD3 implicates conformational selection and allostery**

**Nicholas Spellmon ^1,#^, Xiaonan Sun ^1,#^, Wen Xue ^1^, Joshua Holcomb ^1^, Srinivas Chakravarthy ^2^, Weifeng Shang ^2^, Brian Edwards ^1^, Nualpun Sirinupong ^3^, Chunying Li ^4,^* and Zhe Yang ^1,^***

**^1^** Department of Biochemistry and Molecular Biology, Wayne State University School of Medicine, Detroit, Michigan, USA

**^2^** Center for Synchrotron Radiation Research and Instrumentation and Department of Biological and Chemical Sciences, Illinois Institute of Technology, Chicago, Illinois, USA

**^3^** Nutraceuticals and Functional Food Research and Development Center, Prince of Songkla University, Hat-Yai, Songkhla, Thailand

**^4^** Center for Molecular and Translational Medicine, Georgia State University, Atlanta, GA, USA

**^#^** These authors contributed equally to this work.

*** Correspondence:** Email: zyang@med.wayne.edu; cli19@gsu.edu; Tel: +1-313-577-1294;
Fax: +1-313-577-2765.


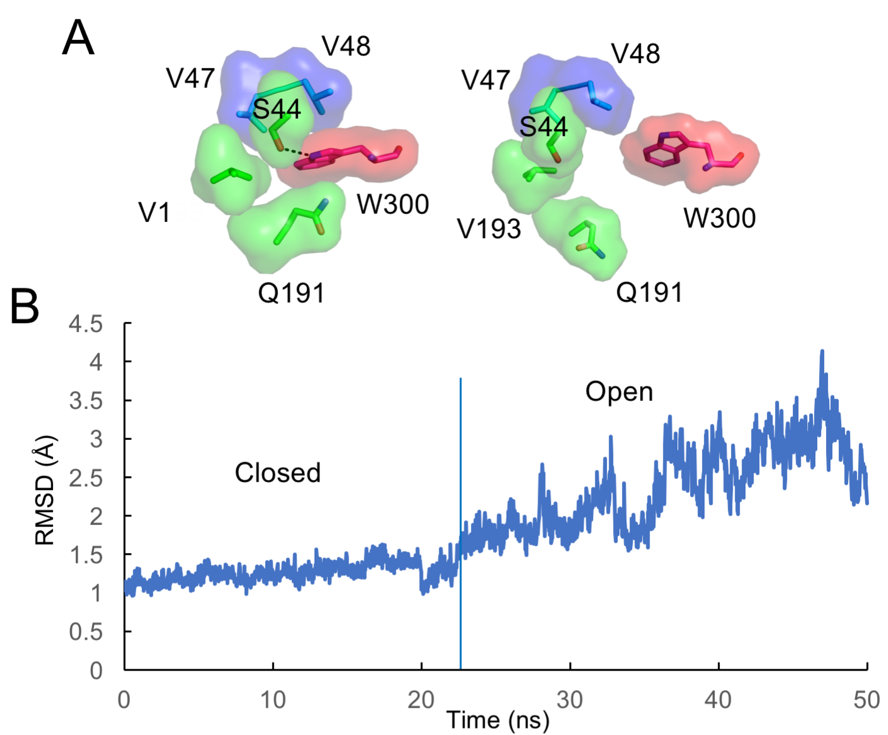


**Figure S1.** New open conformation of SMYD3. (A) W300 interaction in the closed (left) and open (right) states. Hydrogen bond is illustrated as a black dash line. (B) Backbone RMSD during the simulation. RMSD was calculated relative to the crystal structure.


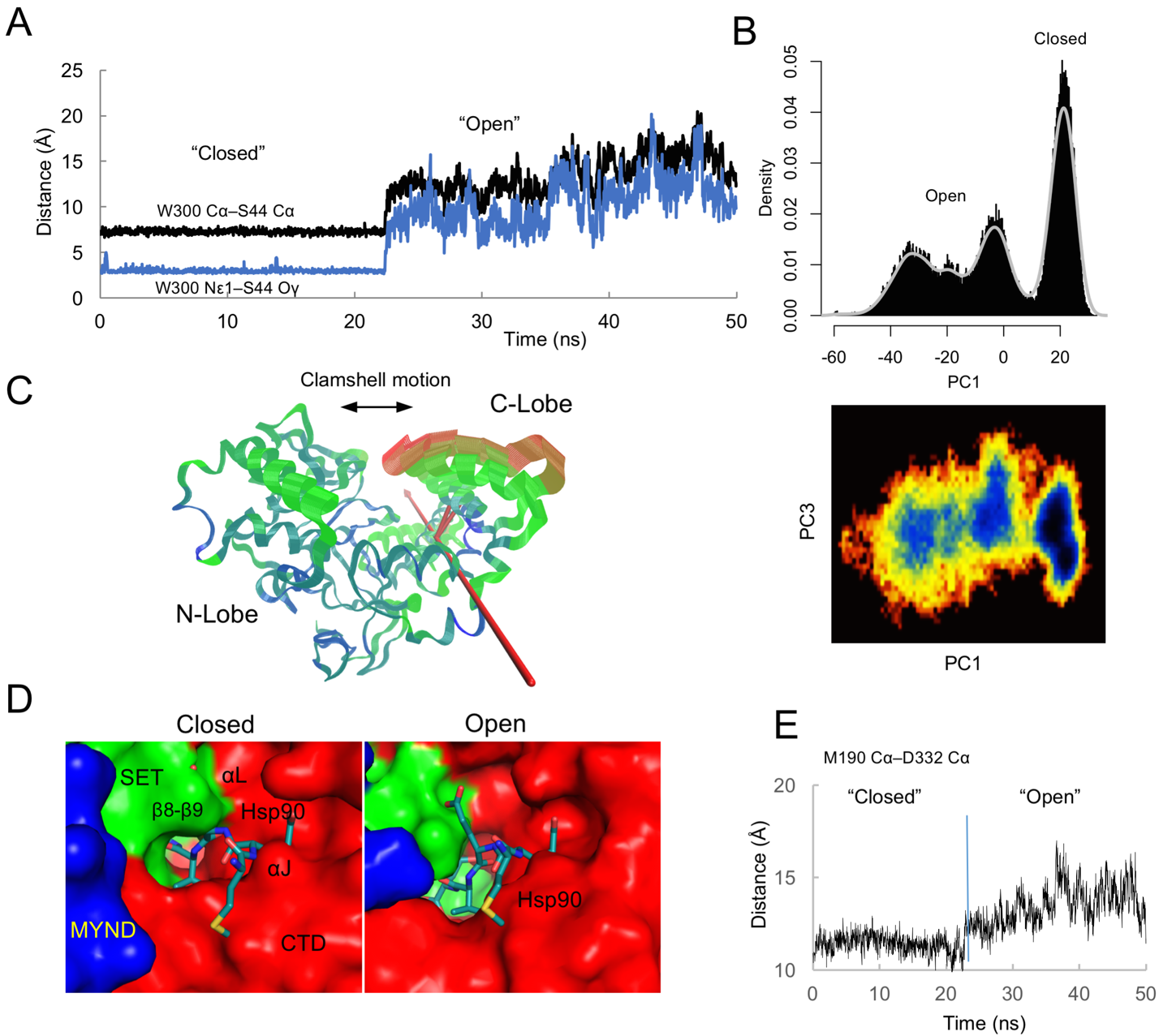


**Figure S2.** Conformational transition leading to a ligand-binding-capable conformer. (A) Distance fluctuation of W300–S44 during the simulation: Cα–Cα (black line) and Nε1–Oγ (blue line). (B) Histogram of the distribution of conformers along the PC1 axis (top) and on a selected PC plane (bottom). The latter was produced by the program Carma, and color scale from red, yellow, to blue depicts low to high frequencies. (C) Visualization of the motions along PC1. The most dissimilar structures are depicted by thicker coils. The interpolated structures produced by Bio3D are shown by thinner coils. Color scale from blue, green, to red depicts low to high atomic displacements. The orientation of the molecule differs by ~90° from that in Fig. 1D. (D) A model of SMYD3­–Hsp90 interaction. Left, the closed state; right, open state. (E) Change in the distance between M190 and D332 depicts the distance fluctuation between the β8–β9 hairpin and Hsp90-binding site.


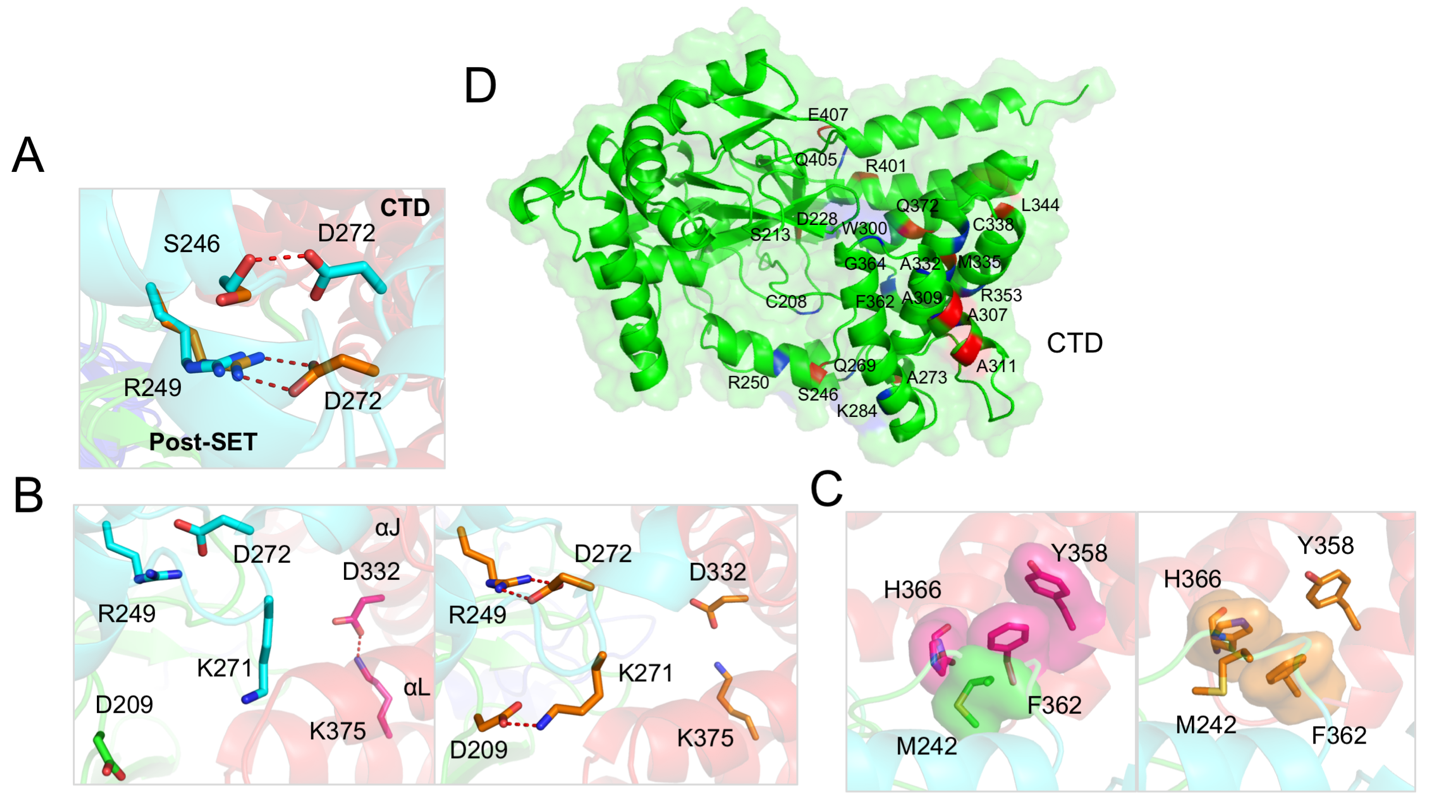


**Figure S3.** Distinct structural characteristics of the closed and open states. (A) D272 involved in conformational-state specific hydrogen bonds. Residues of the closed state are colored cyan and open state orange. Hydrogen bonds are illustrated as red dash lines. (B) Salt-bridges with the strongest correlation with the conformational states. Left, the closed state; right, open state. Salt-bridges are illustrated as red dash lines. (C) F362 interacting environments in the closed (left) and open (right) states. (D) Residues with conformational state-correlated SASA changes. Blue indicates residues being more buried in the open state than closed state and red more exposed.


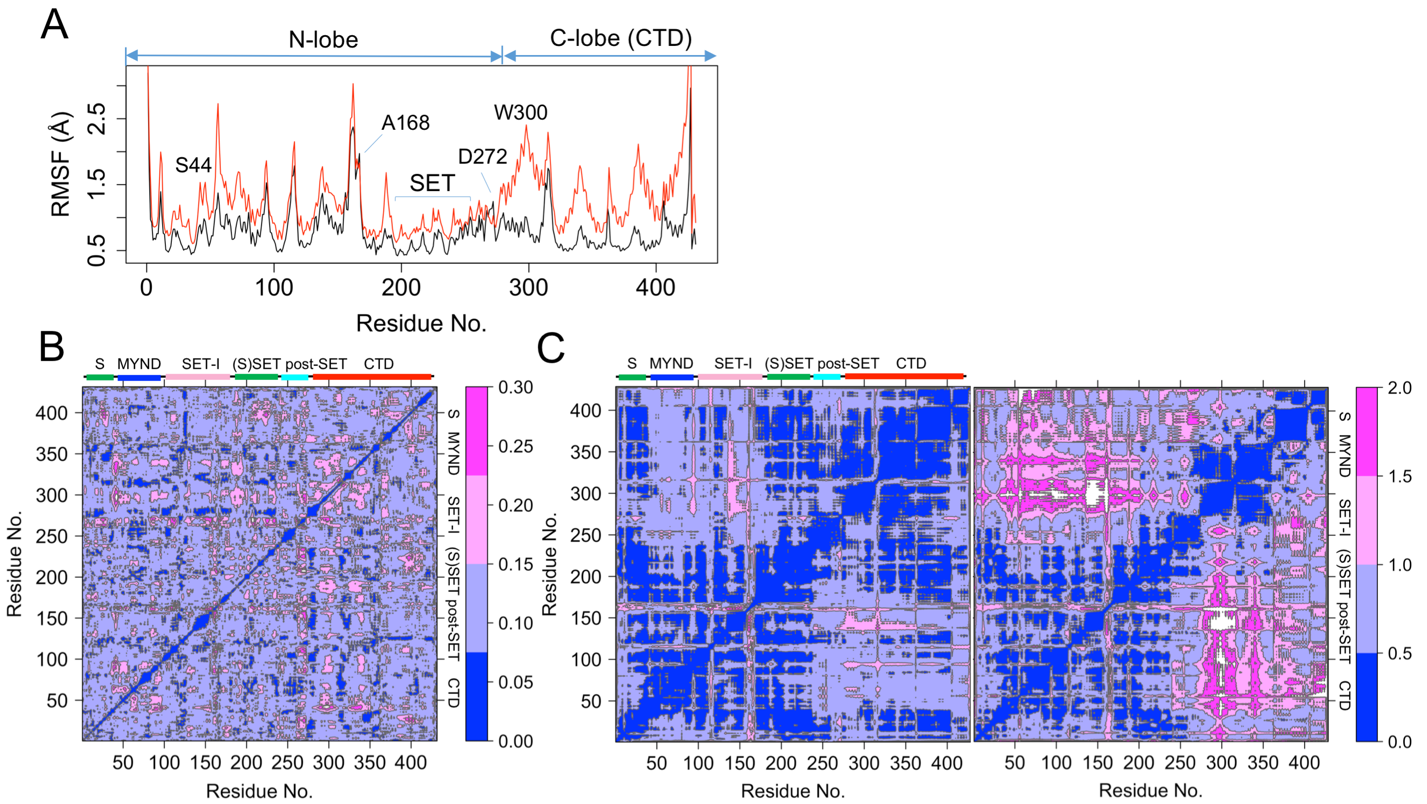


**Figure S4.** Different dynamical characteristics. (A) Root mean square fluctuation (RMSF) of Cα atoms during the simulation. Black line, the closed state; red line, open state. (B) Inter-residue RCC deviation map. Domain structures of SMYD3 are indicated on the top and right of the maps. Color scale from blue to magenta depicts small to large RCC standard deviations. (C) Inter-residue distance deviation map. Color scale from blue to magenta depicts small to large distance deviations. White indicates the deviations of > 2.0 Å.


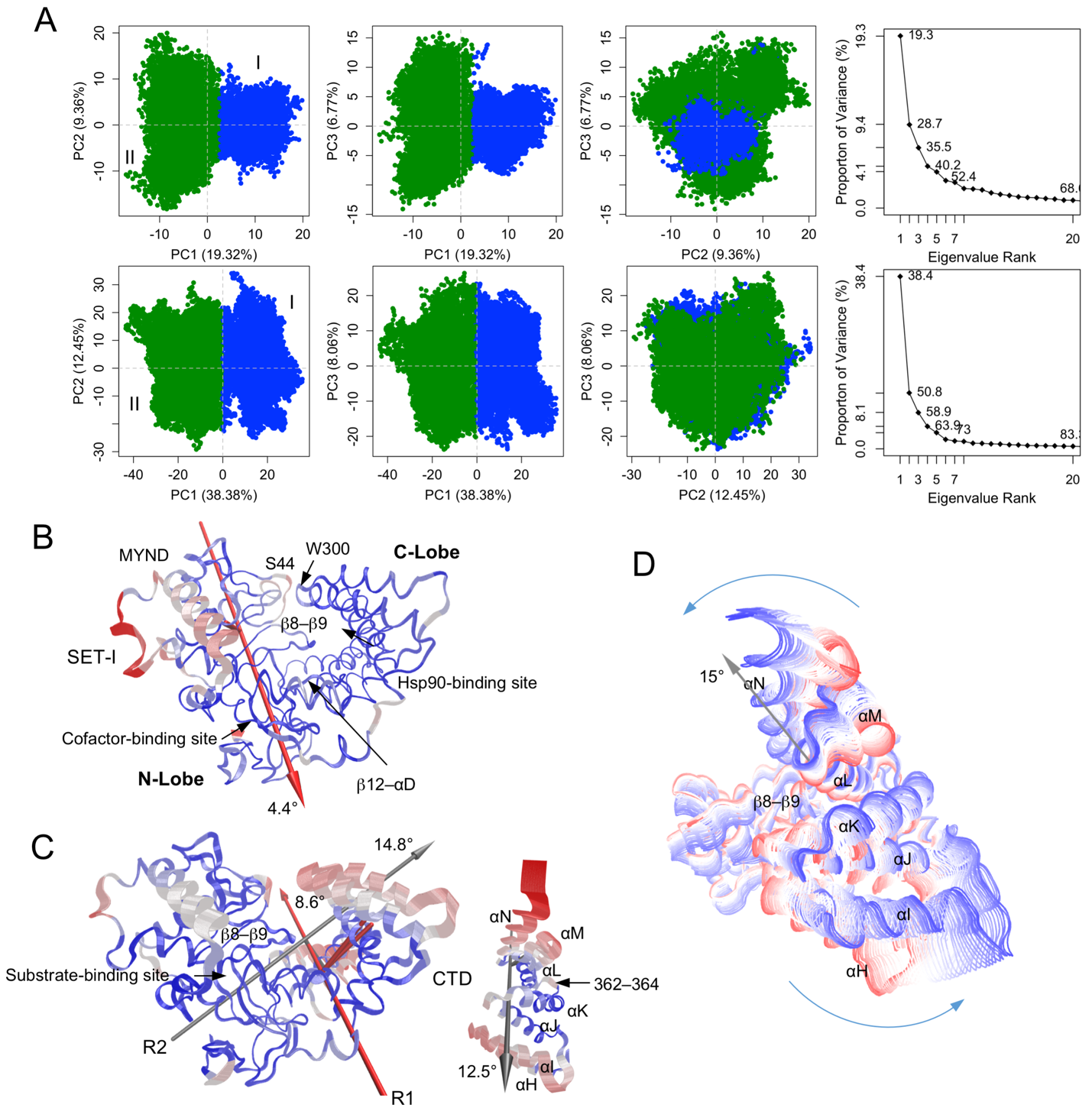


**Figure S5.** Conformational substates. (A) Principle component analysis of the closed (top) and open (bottom) states. Left three, projection of the trajectory onto the planes formed by the first three principle components. Conformers are colored according to the *k*-means clustering: substate I, blue; II, green. Rightmost, scree plot showing the proportion of variance against its eigenvalue rank. (B) Visualization of closed- and (C) open-state motions along PC1. Color scale from blue, green, to red depicts low to high atomic displacements. The right panel of C shows intra-domain motions of the CTD. (D) Conformational transition pathway revealed by targeted molecular dynamics simulation. The trajectory is colored from red to blue in order of time.


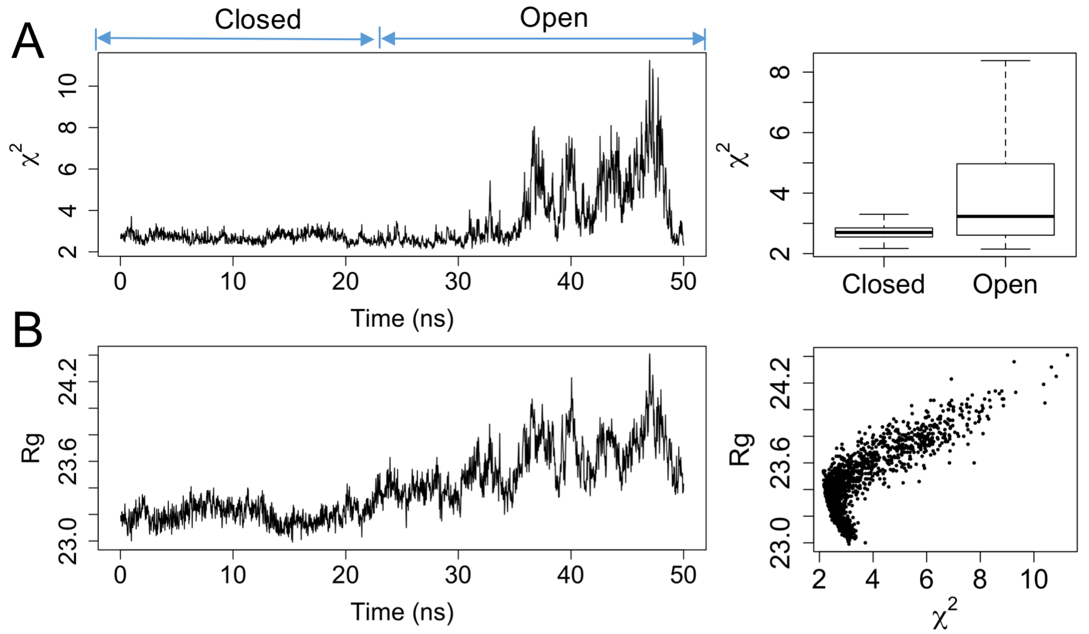


**Figure S6.** Small-angle X-ray scattering. (A) Fitting of the MD trajectory to SAXS data. Left, *χ*^2^ as a function of time; right, a boxplot of the closed- and open-state *χ*^2^. (B) Rg of the trajectory as a function of time (left) and a scatter plot of the trajectory *χ*^2^ and Rg (right).

**Table S1.** Correlation of Psi and Phi with conformational states.

| **Residue** | ***r_psi_^*^*** | **δ_psi_ (°)^#^** | ***r_phi_^*^*** | **δ_phi_ (°)^#^** |
| --- | --- | --- | --- | --- |
| R14 | ^$^ |  | 0.528 |  |
| E57 |  |  | 0.566 | 34.5 |
| C62 | 0.527 |  |  |  |
| S63 | 0.512 |  |  |  |
| Q64 | 0.537 | 30.5 |  |  |
| P167 | 0.740 | 109.9 | 0.679 |  |
| A168 | 0.956 | 50.0 | 0.895 | 135.6 |
| F169 | 0.725 |  | 0.614 |  |
| D170 | 0.680 |  |  |  |
| S207 | 0.543 |  |  |  |
| C208 | 0.654 |  | 0.522 |  |
| P210 | 0.666 |  |  |  |
| R227 |  |  | 0.551 |  |
| L243 | 0.821 | 30.8 |  |  |
| M244 |  |  | 0.702 | 54.5 |
| E260 | 0.929 | 32.7 |  |  |
| C263 |  |  |  | 32.5 |
| K271 | 0.931 | 35.4 |  |  |
| N316 | 0.604 |  |  |  |
| F362 | 0.983 | 48.9 | 0.839 |  |
| P363 | 0.658 | 174.1 | 0.653 |  |
| G364 | 0.750 | 126.1 |  |  |
| S365 | 0.729 | 67.1 |  | 39.8 |
| H366 |  |  |  | 44.3 |
| R401 |  |  | 0.509 |  |
| H404 | 0.772 |  |  |  |
| E407 | 0.713 | 54.8 |  |  |
| H408 |  |  | 0.676 | 40.4 |
| A427 | 0.642 | 113.3 |  |  |

^*^*r_psi_ and r_phi_*, correlation coefficients between torsion angles and conformational states.

^#^δ_psi_ and δ_phi_, mean absolute differences of torsion angles between the closed and open states.

^$^Empty cells indicate *r* < 0.5 or δ < 30**°.**
